# Increased Set Shifting Costs in Fasted Healthy Volunteers

**DOI:** 10.1371/journal.pone.0101946

**Published:** 2014-07-15

**Authors:** Heather M. Bolton, Paul W. Burgess, Sam J. Gilbert, Lucy Serpell

**Affiliations:** 1 Division of Psychology and Language Sciences, University College London, London, United Kingdom; 2 South London and Maudsley NHS Foundation Trust, Bethlem Royal Hospital, Beckenham, United Kingdom; 3 North East London NHS Foundation Trust, Porters Avenue Health Centre, Dagenham, Essex, United Kingdom; University of Bologna, Italy

## Abstract

We investigated the impact of temporary food restriction on a set shifting task requiring participants to judge clusters of pictures against a frequently changing rule. 60 healthy female participants underwent two testing sessions: once after fasting for 16 hours and once in a satiated state. Participants also completed a battery of questionnaires (Hospital Anxiety and Depression Scale [HADS]; Persistence, Perseveration and Perfectionism Questionnaire [PPPQ-22]; and Eating Disorders Examination Questionnaire [EDE-Q6]). Set shifting costs were significantly increased after fasting; this effect was independent of self-reported mood and perseveration. Furthermore, higher levels of weight concern predicted a general performance decrement under conditions of fasting. We conclude that relatively short periods of fasting can lead to set shifting impairments. This finding may have relevance to studies of development, individual differences, and the interpretation of psychometric tests. It also could have implications for understanding the etiology and maintenance of eating disorders, in which impaired set shifting has been implicated.

## Introduction

Cognitive flexibility is an executive function that allows us to behave adaptively in accordance with shifting goals and intentions. Neuropsychological studies have operationalized this flexibility using a variety of set shifting (or task switching) paradigms. In such paradigms, participants must, on certain trials, update the rules they use to determine the correct response to each stimulus. Performance can then be compared between shift trials (where task rules differ from the previous trial) and repeat trials (where the task is repeated). Performance is typically poorer on shift than repeat trials [1], [2]. fMRI studies have indicated that set shifting abilities are linked to particular neural structures, such as the medial prefrontal cortex and anterior cingulate cortex [3]. Furthermore, the “shift cost” has been found to be enhanced, compared with healthy young adults, in older adults, participants with acquired brain damage, and a variety of clinical populations [4]–[6].

Individuals with Anorexia Nervosa (AN) constitute one such population. AN is a severe and chronic eating disorder with the highest mortality rate of any psychiatric illness [7], [8] and treatment efficacy is limited [9], [10]. Cognitive inflexibility has been cited repeatedly as a significant contributing factor in the maintenance of AN [11], [12], manifesting as a rigid focusing of attention on food and weight, obsessive traits and a perfectionistic, inflexible personality style [13], [14].

Numerous studies have demonstrated that patients with AN are impaired on a range of set shifting tasks relative to healthy controls [13], [15]–[17] with corresponding abnormalities in frontal brain areas [18]. This cognitive rigidity cannot be explained as reflecting a general intellectual deficit or diminished information processing. Individuals with AN tend to have higher than average IQs [19], suggesting a relatively specific impairment, hindering the ability to change responses in accordance with shifting contingencies [20].

Although the existence of cognitive rigidity has been well-replicated in studies of currently ill AN patients, and has been cited as a maintaining factor and a barrier to psychological treatment [21], there is some debate over the extent to which this impairment is the result of a temporary starvation-induced state (a result of the physiological effects of fasting), or represents a more enduring impairment.

It has been argued that set shifting deficits in AN exist at least partially independently of nutritional status [11] and may have genetic and neurobiological underpinnings [15], [22]–[24]. In some studies, set shifting deficits have been shown to persist in patients following recovery from AN [17], [25]–[29]. However, others have reported that such deficits improve with recovery [30], [31] and are related to the degree of fasting [32], [33]. In a preliminary study, Merwin et al. [34] report that psychological flexibility improved in parallel with symptom remission from anorexia nervosa. Danner and colleagues [35] caution that there are large individual differences in the degree of impairment shown in AN and that set shifting difficulties may only occur in a subset of patients. Furthermore, most studies in adolescents with AN show no set shifting abnormalities [36]; [37], suggesting the difficulties may be related to chronic starvation. Whilst some neuroimaging studies have suggested that abnormalities persist following weight restoration [38]–[40], others have demonstrated that the brain changes observed in patients with AN can normalise with full weight restoration [41]; [42].

Given this continued debate over whether set shifting is a cause or a symptom of AN, and some criticisms directed at existing research as lacking control of confounding factors [20], the present study attempted to provide some greater clarification of the potential interaction between starvation and set shifting. By investigating the impact of temporary food restriction on the baseline set shifting ability of healthy volunteers, we aimed to clarify whether set shifting deficits could be influenced by the effects of food restriction.

Previous studies have examined the impact of food restriction or fasting on cognition and behaviour. In these, fasting has led to observable perseverative behaviour in humans and laboratory animals [30], [43]–[45]. Furthermore one study [46] reported that after fasting for 24 hours, healthy participants' brains showed differential patterns of activation in the insula, dorsolateral prefrontal cortex and inferior occipito-temporal cortex compared to a satiated state, in response to food-related stimuli.

A systematic review of the literature examining the impact of experimental starvation on cognition in general [47] found 4 studies which included a measure of cognitive flexibility. Three studies used a Stroop task to measure flexibility; two of these 48 & 49 found non-significant trends towards a decrement in performance amongst fasting participants, whilst the third [50] found no differences in cognitive flexibility. However, the use of the Stroop task may be criticised as it appears to measure a number of different aspects of executive function, not just cognitive flexibility [51]. Piech and colleagues [45] examined executive functioning in terms of set-shifting ability. Using a modified Wisconsin Card Sorting Test (WCST), they found that fasted participants were slower and less accurate at set shifting, but only when they were primed by viewing food images.

The current study aims to establish whether performance on a novel measure of set shifting, is impacted by fasting. Given recent evidence of differential neural responses in eating disorders to food and non-food stimuli [52], [53] we decided to develop a novel set-shifting task which included both food and non-food images. Furthermore, we aimed to assess the extent to which anxiety and depression may moderate any deficits in set shifting.

If fasting is shown to have a direct effect on cognitive flexibility, this will raise important questions to be answered for our understanding of AN. Firstly, it will be important to determine the extent to which the set shifting deficit characteristic of AN is accounted for by the day-to-day effects of severe calorie restriction or fasting. Furthermore, it will be important to determine whether food deprivation interacts in some way with a pre-existing tendency to rigidity [54], exaggerating inflexibility in an individual with rigid traits. If this is the case then this might inform a theory of AN that accounts for the role of fasting in the maintenance of AN (as in the model proposed by Treasure and Schmidt [14]) and eventually leads to more effective clinical treatment.

By using a within subjects design, whereby participants are randomised to be fasted on one occasion and satiated on the other, it is possible to control for many of the individual differences which might also affect set shifting performance. In any study examining rigidity in AN, it is important to control for the impact of low mood as AN shares a high comorbidity with depression [55] and there is an extensive evidence base demonstrating that depression is linked to weak set shifting [56]–[58]. Of particular note, Wilsdon and Wade [28] established that set shifting ability in patients with AN was mediated by depression scores (see also [59]).

The present study was designed to explore whether set shifting difficulties emerge under conditions of fasting, while accounting for symptoms of depression. A novel rule change paradigm was devised for the current study in order to measure set shifting ability in a controlled manner. The paradigm was intended to allow a comparison between trials in which participants were required to shift their mental set, versus trials in which no set shift was required. The difference in response time (RT) between switch and repeat trials was used as a measure of set shifting ability, assessed under conditions of fasting and satiety. Furthermore, both food and non-food stimulus items were used in the task, to investigate potential effects of food cues on set shifting ability. The study exclusively recruited women since AN is significantly more common in women [60] and women may be more sensitive to the effects of short-term fasting than men [46].

### Hypotheses

1. Mean RTs will be slower on trials where a shift of set is required compared to non-shift trials, and this shift cost will be exacerbated by fasting (i.e. on trials which require a shift of set, fasting will increase the shift cost relative to when switches are made under satiated conditions).
2. In line with previous studies where food-related stimuli compromised ability, we predict that reaction times will be slowed on trials where the stimuli consist of food items compared to non-food items, particularly in the fasting condition.
3. Elevated depression, perseveration and eating disorder symptoms, assessed by relevant questionnaire measures, will each be associated with impaired set shifting ability.

## Method

### Ethics

Ethical approval was obtained from the University College London Research Ethics Committee (reference 1699/001). All participants provided informed written consent before taking part.

### Participants

Power analysis was conducted using G*Power [61], based on a medium effect size, as reported by Wilsdon and Wade [28] in their examination of WCST perseverative errors made by AN patients. It was calculated that 52 participants would be required in order to achieve 80% power. Sixty adult female volunteers were recruited through a poster campaign. Each received £15 compensation for their time. Participants were unaware of the specific aims of the study, although they understood that it sought to contribute to the understanding of eating disorders. Eligibility requirements included not currently receiving treatment for a medical condition, not being pregnant or diabetic, having normal or corrected to normal vision and fluency in English. Handedness was not assessed as part of the recruitment criteria.

### Design

The study adopted a within-subjects repeated measures design comprising two sessions spaced approximately one week apart. All participants were required to abstain from eating or drinking (other than water) for 16 hours prior to one session (fasted condition) and to eat regular meals for the other (satiated condition). The order of these sessions was randomised. At each session, participants undertook the rule change task and completed self-report questionnaires.

### Measures

#### Diary ratings

Several days prior to each testing session, participants were sent an email containing a self-report diary measure. They were required to print this out and bring it with them to the testing session, after filling it in at five set time points (6pm on the evening before testing, 11pm, 8am or on waking, 11am, and on arrival at the testing session). This required ratings of hunger, food preoccupation, mood and irritability, using a Likert scale ranging from 1 (not at all) to 7 (very much so).

#### Questionnaires

At the fasted session participants completed the Hospital Anxiety and Depression Scale (HADS) [62]. At the satiated session, participants completed the HADS, the Persistence, Perseveration and Perfectionism Questionnaire (PPPQ-22 [63] and the Eating Disorder Examination Questionnaire 6.0 (EDE-Q6) [64]. The PPPQ-22 is a 22-item measure, divided into 3 subscales (persistence, perseveration and perfectionism) and has been shown to have adequate test-retest reliability and internal consistency [63]. The EDE-Q6 is a self-report version of the Eating Disorder Examination [65] which is proven to be effective in the identification and assessment of disturbed eating and displays good internal consistency [66].

#### Rule change task

**Figure 1:**
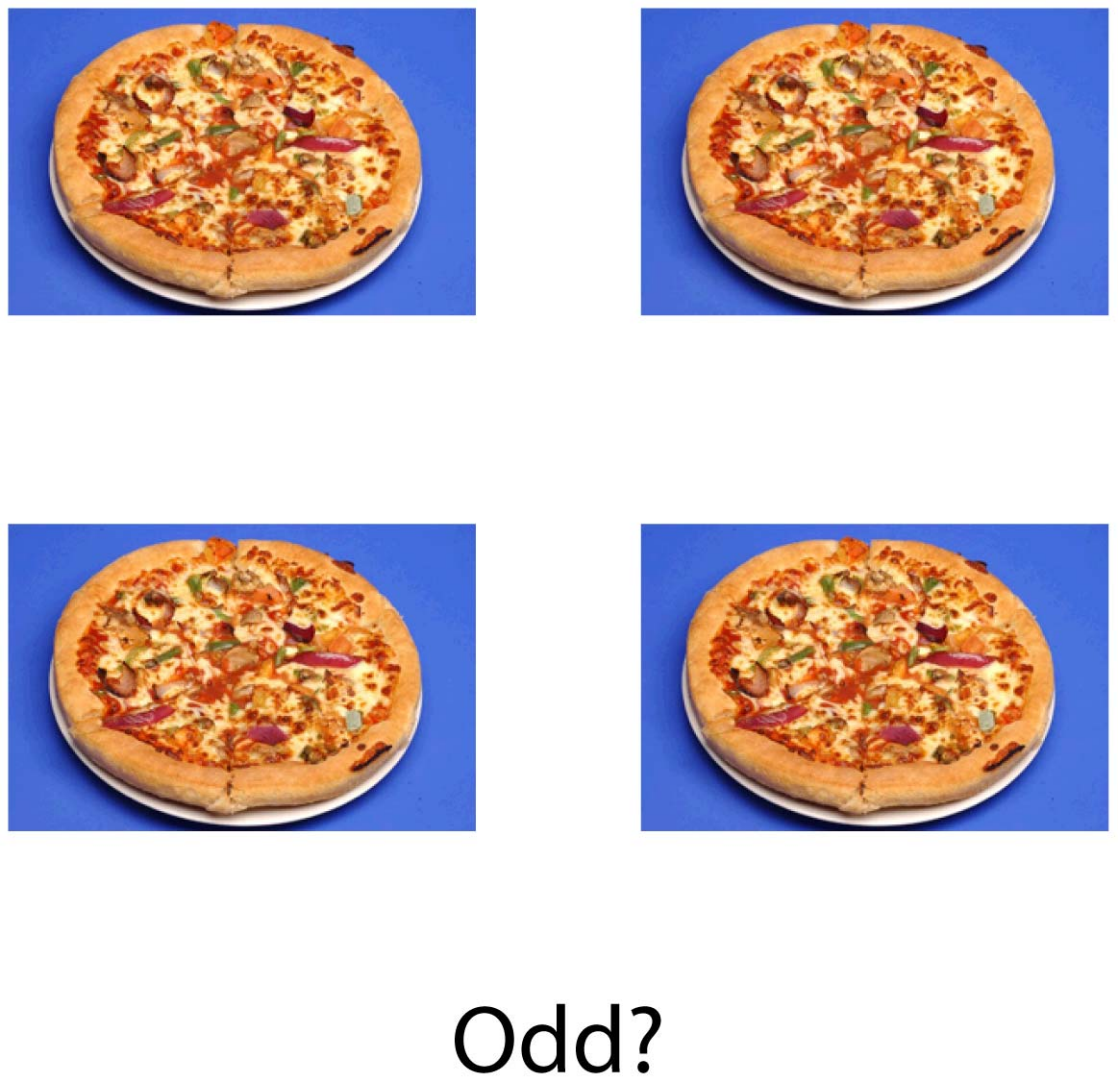
Example trial of the rule change task.

The rule change (RC) task was administered on a laptop using the Cogent toolbox (http://www.vislab.ucl.ac.uk/cogent.php) running under Matlab version 6.5 (MathWorks). Figure 1 shows an example of the experimental stimuli. On each trial, a set of identical photographs was presented in the centre of the screen in a subitized fashion (as on the face of a die) in conjunction with a written question. The question took one of four forms (*Odd?*, *Even?*, *High?* or *Low?*), and required participants to press one of two keys to answer either yes or no, in accordance with the number of pictures on screen. At each presentation the number of photographs on the screen varied from between one and six. If either one, two or three pictures appeared on screen, the question “*Low?*” would require a “Yes” response whilst the correct response to “*High?*” would be to press the “No” key. One, three or five pictures should elicit a press of the “Yes” key to the question “*Odd?*” and “No” to “*Even?*”, and vice versa with two, four or six pictures. The question periodically changed without warning (with a 33% probability on each trial), requiring a shift of mental set. A low probability of shift was chosen in order that participants could not anticipate a shift in advance. On this basis, each trial was classified as either a “shift” trial or a “stay” trial. Shifts took the form of both inter- (e.g. “*High?*” to “*Even?*”) and intra-dimension (e.g. “*High?*” to “*Low?*”) changes, with equal probability. On each trial the stimuli remained on screen until a response key was pressed, followed by a random delay of 250–500 ms and then the next set of stimuli. No feedback was provided about accuracy of responses.

Photographic stimuli were taken from a database created for neuropsychological studies of AN [46], [53]. Each photograph showed either a foodstuff or an inedible item, forming a collection of 118 individual stimuli. Foods consisted of sweet and savoury high-calorie items (e.g. a pizza or a donut) and were photographed on white plates over a blue background. Inedible items were household objects (e.g. a ball of string) which were presented on white circular shapes resembling the plates used in the food pictures, with the same blue background. On each trial there was a 50% probability of a food stimulus or an inedible stimulus being shown.

The computer task was presented on a laptop with a standard keyboard, positioned approximately 60 cm from the seated participant.

### Experimental procedure

Following randomisation for the order of conditions (fasted vs. satiated first), each participant was informed of the particular order of their two sessions by e-mail and was given written instructions for fasting. They were instructed to stop eating at least 16 hours prior to the fasted session and in each case this involved fasting overnight. Participants were encouraged to consume water during the period of fasting, but were required to abstain from alcohol and sugary drinks. In order to ensure compliance with the instructions, participants were informed that their urine would be tested for ketones (a by-product of fasting) at each session.

At each session the diary measure was collected and then the experimental task was administered, followed by the questionnaires and urine test. Participants were presented with written instructions for the task and underwent a practice block of 20 trials followed by 4 blocks of 100 trials. They were instructed to respond as quickly and as accurately as possible, using their dominant hand. At the satiated session, demographic measures were collected and measurements of weight and height were taken using digital scales and a portable stadiometer, for the purposes of calculating body mass index (BMI). At the fasted session, participants were asked to report the length of time for which they had fasted and to rate their subjective ease of fasting on a 5-point scale (1 =  easy, 5 =  hard).

## Results

### Characteristics of the sample

The mean age of the sample was 27.4 years (S.D. = 7.41; range: 19–55). The mean BMI was 22.3 kg/m^2^ (S.D. = 2.9; range: 17.8–29.3) and all participants' BMIs fell above the 17.5 kg/m^2^ clinical cut-off for AN (WHO, 1992). Age and BMI did not significantly correlate (r = .254, p = .059). Thirty-six participants (60%) were working professionals, with the remainder either undergraduate or postgraduate students. Seventy-seven percent of the sample was White and the remainder Asian (8%), Chinese (7%), Black (5%) and mixed race (3%).

Participants reported fasting for an average of 16.67 hours (S.D. = 1.12; range 15.25–21). The mean subjective ease of fasting was rated at 2.42 (S.D. = .85; range 1–5; where 1 =  easy, 5 =  difficult). There were no significant correlations between BMI, subjective ease of fasting and reported length of fast (*p*>.576). Two participants admitted to having eaten during the period in which they were expected to be fasting and their data were excluded from further analyses, leaving a total sample of n = 58.

The urinalysis was not sensitive enough to detect ketones as a longer period of fasting is required before ketones are produced. Nevertheless, as participants believed that urine testing would reveal the extent of their fasting, it served as a tool to ensure compliance with fasting requirements.

Forty-five participants (77.6%) returned both sets of diary measures (see Table 1). Paired samples t-tests were carried out on these diary ratings, comparing ratings at time 1 (T1; the evening before testing) to time 5 (T5; arrival at the testing session). The subjective feeling of hunger significantly increased during the starvation period (mean hunger ratings at T1 = 2.87, and T5 = 4.9; t = 6.27, df = 44, p<.001, d = 1.9). The reverse effect occurred in the satiated condition, as mean hunger levels at T5 were significantly less than T1 (T1 = 2.80, T5 = 2.20; t = 2.20, df = 44, p = .033, d = .66).

**Table 1:** Mean diary ratings during fasted and satiated conditions.

	Fasted		Satiated	
	T1 (day prior to testing)	T5 (immediately prior to testing)	T1	T5
Hunger	2.87***	4.93***	2.80*	2.20*
	(SD = 1.93)	(SD = 1.59)	(SD = 1.65)	(SD = 1.38)
Food preoccupation	2.91***	4.49***	2.75*	1.98*
	(SD = 1.59)	(SD = 1.60)	(SD = 1.43)	(SD = 1.07)
Mood	2.38*	2.91*	2.53	2.27
	(SD = 1.13)	(SD = 1.18)	(SD = 1.08)	(SD = 1.07)
Irritability	2.02*	2.96*	1.91	1.64
	(SD = 1.29)	(SD = 1.59)	(SD = 1.26)	(SD = 1.09)

SD  =  standard deviation

^*^  =  significant difference between T1 and T5 (p<.05)

^***^  =  significant difference between T1 and T5 (p<.001)

Fasting also led to worsening in subjective mood across time (T1 = 2.38, T5 = 2.91; t = 2.63, df = 44, p = .012, d = .79, where 1 =  better mood), an increase in irritability (T1 = 2.02, T5 = 2.96; t = 3.30, df = 44, p = .002, d = .99) and an increase in food preoccupation (T1 = 2.91, T5 = 4.49; t = 4.79, df = 44, p<.001, d = 1.44). Under satiated conditions, food preoccupation significantly decreased over time (T1 = 2.75, T5 = 1.98; t = 3.30, df = 44, p = .002, d = .99), while mood did not significantly change (T1 = 2.53, T5 = 2.27; t = 1.60, df = 44, p = .12, d = .48). There were also no significant changes in irritability (T1 = 1.91, T2 = 1.64; t = 1.37, df = 44, p = .18, d = .41) across time in the satiated condition.

### Questionnaire data

Table 2 shows the mean HADS scores for the sample. In relation to healthy population norms [67], the mean level of anxiety within the sample was comparable, but the prevalence of depression symptoms was lower than conventional levels found in community samples. Neither age nor BMI significantly correlated with HADS-anxiety (p>.244) or HADS-depression (p>.211).

**Table 2:** Mean anxiety and depression scores on the HADS in fasted and satiated conditions.

	Fasted	Satiated	Normed sample (Crawford et al., 2001)
Anxiety	5.86	6.29	6.14
	(SD = 3.59)	(SD = 3.58)	
Depression	2.17	2.9	3.68
	(SD = 2.46)	(SD = 3.22)	

Mean scores on each of the PPPQ-22 subscales were comparable to a non-clinical population assessed by Serpell et al. [63]. Within the subscales, PPPQ-perseveration did not correlate with either persistence (r = .23, p = .084) or perfectionism (r = .13, p = .350). Persistence and perfectionism were strongly positively correlated (r = .42, p<.001). Neither age nor BMI were significantly correlated with any PPPQ-22 subscales (p>.425).

All subscales of the EDE-Q6 were highly intercorrelated (r>.646, p<.001) and mean group scores were comparable to a non-clinical sample [68]. Neither age nor BMI correlated significantly with any subscales. Although restraint bore no significant relationship to the other measures, weight, shape and eating concern of the EDE-Q6 shared moderate positive correlations with HADS depression (r>.348, p<.008) and PPPQ-perseveration (r>.366, p<.005).

### Experimental task performance

Incorrect responses and trials where RT<150 ms or >3000 ms were excluded from all analyses to eliminate outliers (where participants might have been inattentive or responded prematurely to stimuli). Error rates were generally low (mean 6%), except for 2 participants whose accuracy on the task was comparatively low (>3 S.D. from mean accuracy score). Their data were excluded from the analysis, along with the two participants who had admitted to having eaten during the fasting period, because they did not adequately comply with the experimental instructions, making it difficult to interpret results. Seeing as there were only four participants that did not adequately comply with task instructions – too few for a separate analysis – data from these participants were not considered further. Therefore, n = 56 for the main analysis. There were no significant differences between inter- and intra-question shifts (for instance from *high* to *low*, versus from *high* to *odd*) and so these data were collapsed in the results below.

**Figure 2:**
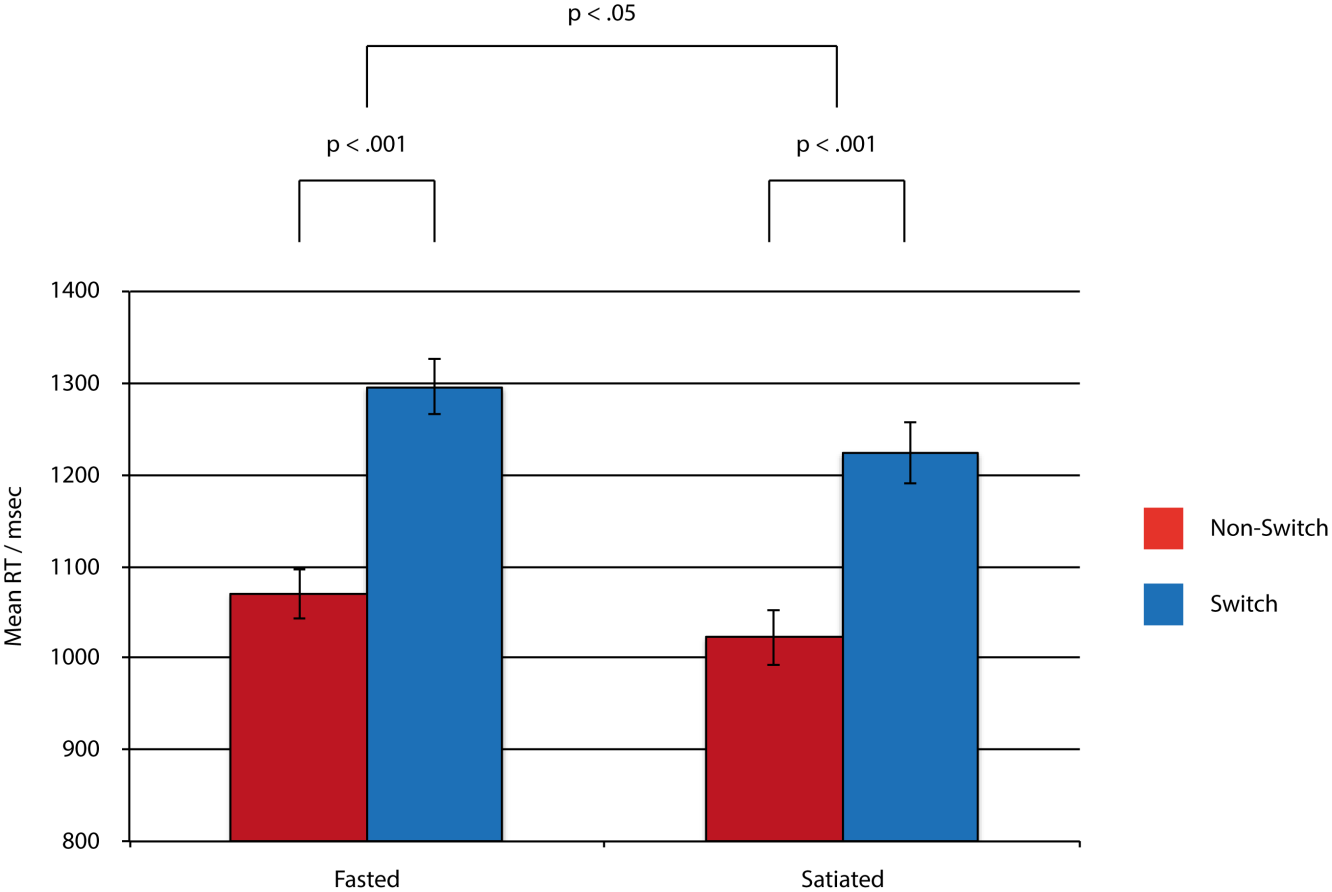
Mean reaction times in the rule change task. Error bars indicate standard errors.

To test the first two hypotheses and explore the effects of fasting, shifting and food stimuli, a general linear model (GLM) was constructed consisting of 3 within-subject factors, each with 2 levels: fasted vs. satiated; shift vs. stay; and food vs. inedible. The GLM investigating RTs indicated a main effect of fast (F (1, 55) = 5.00, *p* = .029, η^2^ = .08), a main effect of shift (F (1, 55) = 403, *p* = <.001, η^2^ = .88) and a significant fast x shift interaction (F (1, 55) = 4.22, *p* = .045, η^2^ = .07). This shows that the shift cost was more pronounced under conditions of fasting. There was no main effect of food pictures (F (1, 55) = .015, *p* = .90, η^2^<.01) and no significant interactions with this factor (F (1, 55)<3.3, p>.08, η^2^<.06). These results are summarised in Figure 2 and Table 3.

**Table 3:** Mean reaction times (RT) and percent accuracy in the rule change task.

	Fasted		Satiated	
	Non-switch	Switch	Non-switch	Switch
RT (msec)	1070	1296	1023	1224
	(SD = 201)	(SD = 228)	(SD = 224)	(SD = 249)
Accuracy (%)	95	92	97	93
	(SD = 3)	(SD = 5)	(SD = 3)	(SD = 5)

Analysis of accuracy data revealed significantly lower accuracy in the fasting (93.6%) than the satiated condition (94.9%; F(1, 55) = 7.5, p = .008, η^2^ = .12). Accuracy was also significantly lower on shift trials (92.7%) than repeat trials (95.9%; F(1, 55) = 91, p<.001, η^2^ = .62). No other effects were significant (F(1, 55)<2.2, p>.14, η^2^<.04).

In order to test the third hypothesis, which predicted that questionnaire scores would relate to task performance, correlational analysis was undertaken. From the rule change task, three variables were entered into the correlation analyses: 1) fast vs. satiated (i.e. overall difference in RT between fasted and satiated sessions); 2) shift cost (i.e. overall difference in RT between switch and repeat trials); 3) fast x shift interaction (i.e. difference in shift cost between fasted and satiated sessions).

Pearson correlations revealed that none of the 3 variables shared significant relationships with subscales of the PPPQ-22 or HADS. Only the EDE-Q6 scale of weight concern significantly correlated with the *fast vs. satiated* variable (r = .348, p = .009). There were no correlations between the 3 variables and any of the demographic measures of BMI, age, length of fast or subjective ease of fasting (p>.19).

## Discussion

This study used a novel set shifting paradigm to examine the influence of short-term fasting on cognitive flexibility in a non-clinical population. In line with our first hypothesis, short-term food deprivation impaired set shifting ability in our healthy sample. Our second hypothesis – that food-related stimuli would impair performance and exacerbate shift costs – was not supported: we found that the content of the stimuli did not have a significant impact on RT. Counter to our third hypothesis, the interaction between fasting and set shifting occurred independently of self-reported symptoms of depression, perseveration and disordered eating, aside from the association with weight concern.

The finding of an interaction between fasting and set shifting is intriguing, especially given the theory that the cognitive inflexibility observed in patients with AN is at least partially due to the biological effects of short-term food restriction [31], [33]. Whilst this study has been conducted within a non-clinical population and thus cannot tell us anything directly about AN, it is noteworthy that even short-term starvation had a significant impact on set shifting.

Although this study aimed to examine the impact of food restriction on cognitive performance in a nonclinical sample, it is important to note that short-term fasting is not equivalent to the chronic undernourishment seen in AN. Chronic starvation leads to neurochemical, metabolic, and structural brain changes in patients with AN [69], [70], some of which may persist after weight restoration [30]. Fasting for sixteen hours is unlikely to lead to such changes. Future research comparing AN patients with healthy controls who had been fasting for at least sixteen hours, as well as with individuals who limit their food intake for other reasons, may help us to understand further whether the changes in performance seen in this study are comparable with the cognitive profile of AN patients. It will also be important to include a range of cognitive tasks to tease out whether the effect is related specifically to cognitive flexibility, or more broadly linked to changes in inhibition or attention.

Even without a predisposition towards cognitive inflexibility, set shifting difficulties might emerge under conditions of fasting triggered by other factors, such as dieting, physical illness, loss of appetite and/or low mood. Following this initial compromise in flexibility during food restriction, cognitive and behavioural rigidity might then be further exaggerated and manifest as symptoms of AN, such as more deliberate food restriction and preoccupation with thinness. Moreover, a perseverative style may also make it difficult for the individual to terminate efforts at food restriction once started.

The weight concern scale of the EDE-Q6 was correlated with a general performance decrement under fasted conditions. Although this was not specific to set shifting, it illustrates that the very individuals who are more concerned over their weight are those whose cognitive processes are most detrimentally affected by food deprivation. Clinically this might indicate that some vulnerability exists in those with weight concern, in that these are people who are likely to be restricting their food intake frequently.

Counter to expectations, self-reported depression scores did not have any significant bearing on task performance. This suggests that depressive symptomatology did not impact upon cognitive flexibility in the sample. However, the levels of reported depression in the sample were below population norms [67] and therefore a sample with a broader range of levels of depression might allow for a more accurate test of the hypothesis that low mood would relate to poorer task performance.

### Clinical implications

The results provide tentative support for the view that short-term fasting may contribute to the expression of an individual's propensity towards rigidity, either exaggerating existing set shifting difficulties or evoking a novel, temporary set shifting impairment. Such an explanation would be in line with the argument that the cognitive deficits in AN are associated with the biological effects of food restriction [31]. It might be that individuals with AN show an abnormally high rate of perseveration in response to fasting and those whose flexible cognitive processes are more resilient to the effects of short-term fasting would be at a lesser risk of developing AN. This could contribute to explanations of why food restriction does not lead to the development of AN in the majority of individuals who diet or miss meals.

Another implication is that fasting may potentially account for some of the rigid thoughts and behaviours observed in patients with AN. If this finding is confirmed in future research, it might support an approach to treatment that focuses on initial nutritional rehabilitation before attempting to address cognitive or emotional factors. This is especially important considering that the cognitive inflexibility inherent in AN reduces the ability to fully engage in therapy [28].

### Limitations of the present study and future research needs

The current study was designed to be exploratory and has generated further hypotheses that merit testing. However, there are several limitations to its design and we acknowledge that these may limit the degree to which we can extrapolate our results. Firstly, we cannot draw conclusions about the more general issue of cognitive processing, (i.e. attention and cognitive and motor flexibility) following fasting, as our measurement was restricted to set shifting specifically. Furthermore, as we did not take physiological or neural measures, we cannot draw any conclusions about the biological processes that might mediate or explain the effects of dietary restriction on cognitive flexibility. The exact mechanisms by which fasting led to increased shift costs is also a matter requiring further investigation. This is true both at psychological and neurophysiological levels. For example, at a psychological level it is unclear how far a fasting-induced set shifting deficit should be understood in terms of distraction/preoccupation with feeling of hunger, difficulty inhibiting inappropriate response tendencies, interpreting and updating task rules, or a combination of these and other factors. At a neurophysiological level, the impact of fasting on regional brain activity and distinct neuromodulatory systems also merits further study.

In addition, we acknowledge that sixteen hours is a relatively short period for fasting. However, other recent studies in the literature have investigated the impact of fasting periods as short as two hours on neuropsychological functioning [71]. Future studies aiming to build on our results might benefit from a lengthier period of starvation in order to maximise the impact of food restriction. Linked to this, it was not possible to fully establish whether participants had adhered to the fasting requirements, other than relying on their belief that urinalysis would reveal non-compliance and the encouragement of honest reporting. However, if any participants ate surreptitiously without disclosing it to the experimenter, and the extent of fasting was underestimated in the analysis, this would make the findings more striking.

It might be argued that using an established measure of set shifting (e.g. the WCST) would have provided more compelling results and allowed for comparison with other clinical studies. However, the ecological validity of such tests has been questioned [72], and it is not clear that they have suitable psychometric properties to detect variation in nonclinical samples, in comparison with the RT measures investigated in the present study. Furthermore, the present task was designed to allow comparison between food and non-food stimuli. Nevertheless, it would be helpful for future fasting studies to include multiple measures of set shifting, including more traditional tasks such as the WCST. Practice effects within the rule change task might have lessened the within-subject differences and therefore it may also be useful for future studies to employ a more complex shifting task to reduce the incidence of practice effects. Clinically, the existence of practice effects may add support to interventions such as cognitive remediation therapy [73] which focus on improving cognitive flexibility, as it suggests that cognitive inflexibility can be overcome with effort under conditions of fasting.

### Conclusions

Given the impact of short-term fasting, it is highly recommended that future studies examining cognition in AN investigate the effects of food restriction in their participant samples. Further studies of healthy controls who have fasted for hours or days would also help to establish a clearer picture of the effects of (at least short-term) food restriction on cognition. Furthermore, an explicit examination of the factors that interact with rigid thinking might also be important.

Given that relatively brief dietary restriction had selective effects on performance of the rule change task (i.e. greater effect on switch than repeat trials), it remains to be explored how individual differences in diet over a longer time period (including a developmental timespan) may impact on performance of specific tasks. This has clear implications for the study of development and individual differences. Furthermore, our results suggest that caution should be used in interpreting results from tests conducted in conditions where dietary restriction is common, for example in a hospital setting.
